# Supplementary material for: A Transfer Learning Approach for Microstructure Reconstruction and Structure-property Predictions
Source: Sci Rep. 2018 Sep 7;8:13461. doi: 10.1038/s41598-018-31571-7 (PMC6128837; doi:10.1038/s41598-018-31571-7)
Supplement: Supplementary file 1 — Supplementary Information [file 41598_2018_31571_MOESM1_ESM.pdf]

Appendices for

A Transfer Learning Approach for Microstructure Reconstruction and Structure-property Predictions

Xiaolin Li[1], Yichi Zhang[2], He Zhao[2], Craig Burkhart[3], L Catherine Brinson[2,4,5], Wei Chen[2,*]

[1]Theoretical and Applied Mechanics Program, Northwestern University, Evanston, IL, 60208
[2]Department of Mechanical Engineering, Northwestern University, Evanston, IL, 60208
[3]Global Materials Science Division, The Goodyear Tire and Rubber Company, Akron, OH 44305, USA
[4]Department of Materials Science and Engineering, Northwestern University, Evanston IL, 60208
[5]currently at Department of Mechanical Engineering and Materials Science, Duke University, Durham NC, 27708
*corresponding author, contact: weichen@northwestern.edu

Appendix I. The plots of two-point correlation functions for the material systems in Fig. 2

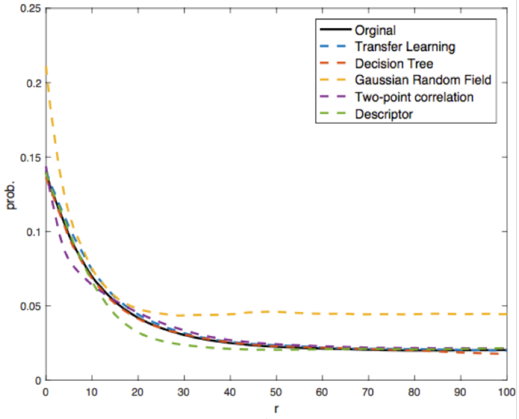

Carbonate

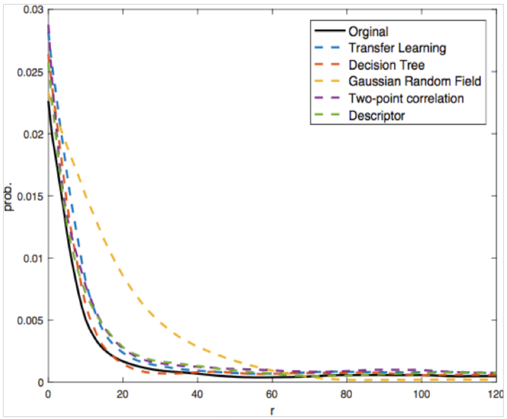

Polymer Comp.

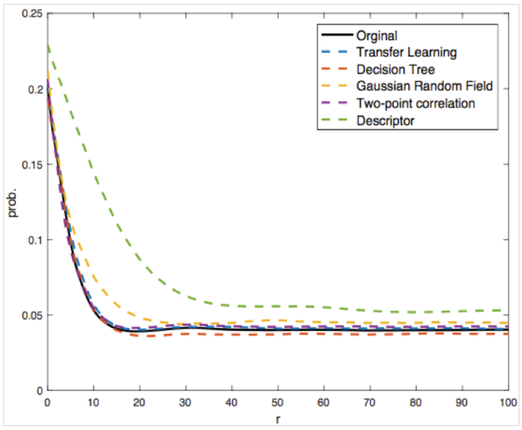

Sandstone

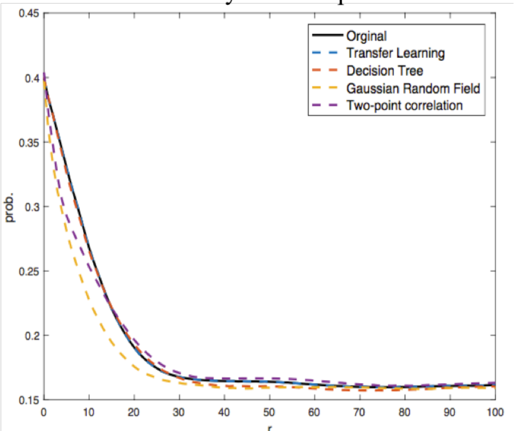

Ceramics

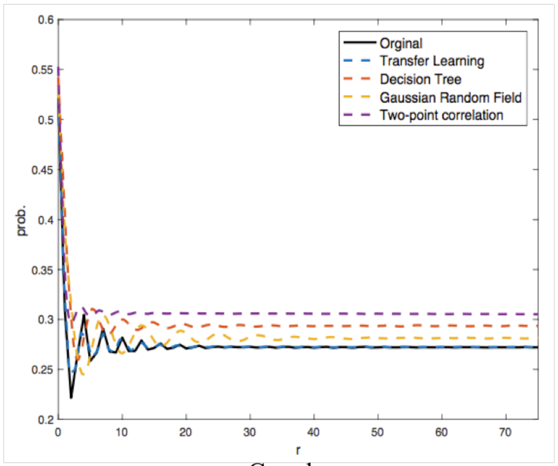

Copolymer

Appendix II. The plots of lineal-path correlation functions for the material systems in Fig. 2

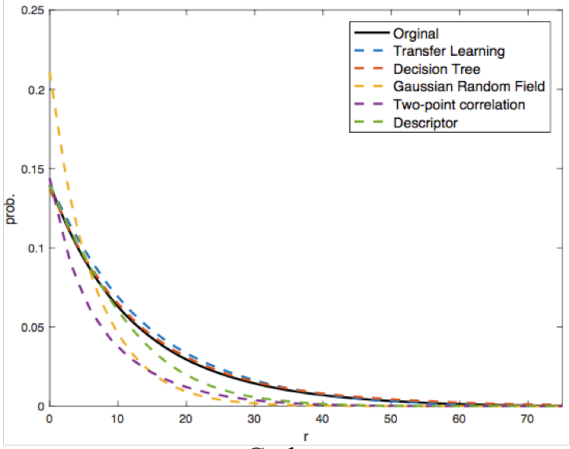

Carbonate

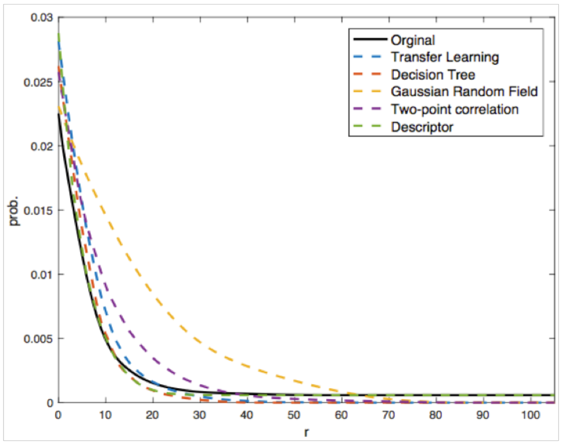

Polymer Comp.

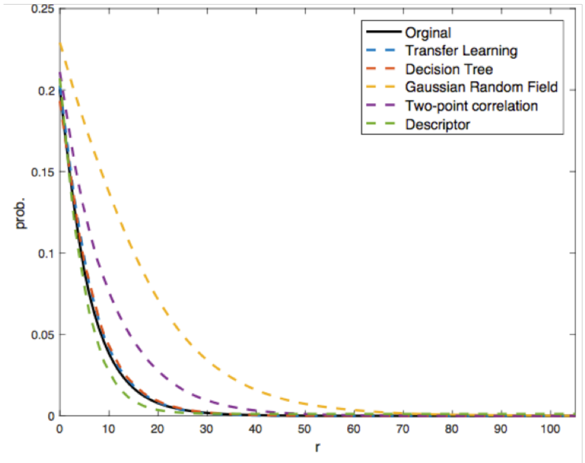

Sandstone

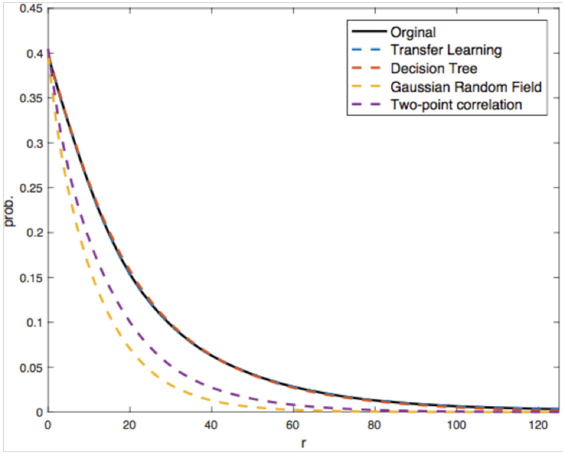

Ceramics

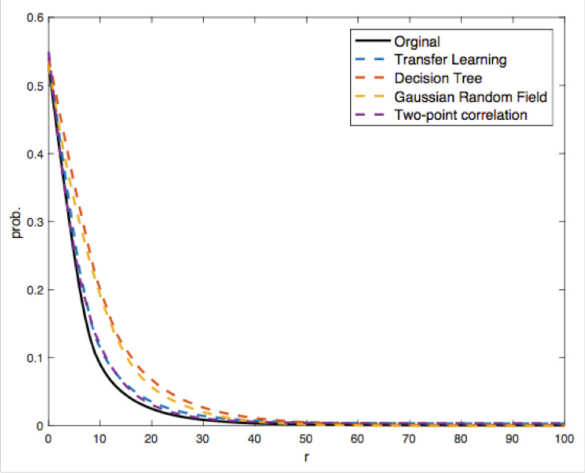

Copolymer
